# A Conserved Peptide Pattern from a Widespread Microbial Virulence Factor Triggers Pattern-Induced Immunity in *Arabidopsis*

**DOI:** 10.1371/journal.ppat.1004491

**Published:** 2014-11-06

**Authors:** Hannah Böhm, Isabell Albert, Stan Oome, Tom M. Raaymakers, Guido Van den Ackerveken, Thorsten Nürnberger

**Affiliations:** 1 Center of Plant Molecular Biology (ZMBP)-Plant Biochemistry, Eberhard-Karls-University Tübingen, Tübingen, Germany; 2 Plant-Microbe Interactions, Department of Biology, Utrecht University, Utrecht, The Netherlands; 3 Centre for BioSystems Genomics (CBSG), Wageningen, The Netherlands; Scottish Crop Research Institute, United Kingdom

## Abstract

Microbe- or host damage-derived patterns mediate activation of pattern-triggered immunity (PTI) in plants. Microbial virulence factor (effector)-triggered immunity (ETI) constitutes a second layer of plant protection against microbial attack. Various necrosis and ethylene-inducing peptide 1 (Nep1)-like proteins (NLPs) produced by bacterial, oomycete and fungal microbes are phytotoxic virulence factors that exert immunogenic activities through phytotoxin-induced host cell damage. We here show that multiple cytotoxic NLPs also carry a pattern of 20 amino acid residues (nlp20) that triggers immunity-associated plant defenses and immunity to microbial infection in *Arabidopsis thaliana* and related plant species with similar characteristics as the prototype pattern, bacterial flagellin. Characteristic differences in flagellin and nlp20 plant responses exist however, as nlp20s fail to trigger extracellular alkalinization in *Arabidopsis* cell suspensions and seedling growth inhibition. Immunogenic nlp20 peptide motifs are frequently found in bacterial, oomycete and fungal NLPs. Such an unusually broad taxonomic distribution within three phylogenetic kingdoms is unprecedented among microbe-derived triggers of immune responses in either metazoans or plants. Our findings suggest that cytotoxic NLPs carrying immunogenic nlp20 motifs trigger PTI in two ways as typical patterns and by inflicting host cell damage. We further propose that conserved structures within a microbial virulence factor might have driven the emergence of a plant pattern recognition system mediating PTI. As this is reminiscent of the evolution of immune receptors mediating ETI, our findings support the idea that there is a continuum between PTI and ETI.

## Author Summary

Eukaryotic host immunity to microbial infection requires recognition systems sensing the presence of potential invaders. Microbial surface structures (patterns) or host breakdown products generated during microbial attack serve as ligands for host immune receptors (pattern recognition receptors) mediating activation of immune responses. Microbial pathogens employ, however, host-targeting effector proteins to establish infection, and the efficiencies of microbial pathogen attack and host defense mechanisms determine the outcome of microbe-host interactions. Necrosis and ethylene-inducing peptide 1 (Nep1)-like proteins (NLPs) from bacteria, oomycetes and fungi are cytotoxic virulence factors (effectors) that trigger plant immunity through toxin-induced host cell damage. Here we show that, in addition, numerous NLPs harbor a characteristic 20-mer sequence motif (nlp20) that is recognized by Brassicacae plant species and perception of which confers immunity to infection by bacterial, oomycete and fungal pathogens. Our findings provide evidence that cytotoxic NLPs are virulence factors that trigger plant immunity by pattern recognition and by inflicting host cell damage. We further conclude that NLPs from prokaryotic and eukaryotic microorganisms and from three organismal kingdoms evoke plant defense. Such an exceptionally wide taxonomic distribution of microbe-derived triggers of immunity has neither been reported before from metazoans nor from plants.

## Introduction

Plants make use of a bipartite immune system to cope with microbial infection [1]. Microbial pattern recognition by host-encoded immune receptors is essential for the activation of plant antimicrobial defenses. Perception by pattern recognition receptors (PRRs) of pathogen-associated molecular patterns (PAMPs) is referred to as PAMP-triggered immunity (PTI) [2], [3]. PTI is an ancient form of plant immunity that provides protection to host non-adapted pathogens, but limited or basal immunity to host-adapted microbes only. In addition, plant-derived damage-associated molecular patterns (DAMPs) are released either by the deleterious activities of secreted microbial enzymes or toxins that activate plant PTI in a PRR-dependent manner [2], [4]. Host-adapted plant pathogens employ effectors to suppress PTI and to establish infection [5]. Co-evolution of hosts and host-adapted microbes has resulted in effector-triggered immunity (ETI), which is dependent on immune receptors recognizing effectors directly or indirectly through sensing effector-mediated manipulations of host targets [1], [3], [6].

Plants recognize a wide range of proteinaceous, carbohydrate or lipophilic PAMPs [2], [7]. In most cases, small epitopes within such patterns provide ligands for plasma membrane-localized PRRs [8], [9]. These ligands are often broadly conserved among microbial species or genera and are not subject to frequent mutations likely because of their vital cellular functions [10]. Well-studied microbe-derived triggers of plant immunity comprise structurally conserved N-terminal regions of bacterial flagellin (flg22) and elongation factor Tu (EF-Tu, elf18) or oligomeric carbohydrate fragments of bacterial peptidoglycans, fungus-derived chitin or oomycete cell wall β-glucans [2], [7]. Plant perception systems for flagellin, peptidoglycans or chitin are rather widespread among plant families, suggesting that these systems are evolutionarily ancient [11]. In contrast, EF-Tu or β-glucan receptors appear to have evolved more recently as perception systems are restricted to members of the Brassicaceae or Fabaceae families only [12], [13]. Likewise, more recently identified *Sclerotinia sclerotiorum*-derived proteinaceous SSCF1 or *Xanthomonas campestris*-derived EMAX are recognized by Brassicaceae only [14], [15]. Moreover, identification of a tomato flagellin perception system that recognizes flagellin epitopes different from flg22 [16], or of a rice receptor that recognizes a central fragment of EF-Tu structurally unrelated to elf18 [17], suggest substantial dynamics in PRR evolution. More systematic studies on PRR distribution patterns among *Arabidopsis thaliana* ecotypes have further revealed that individual pattern recognition specificities might also be lost during evolution [14], [15], [18]. In fact, such ecotype-specific differences in microbial pattern recognition are now increasingly being used to identify novel plant PRRs and to test their phytoprotective potential in crops [14], [19]. Altogether, loss and gain of plant PRRs appears to be a characteristic of plant immunity that is also reminiscent of the dynamics underlying evolution of plant immune receptors mediating microbial ETI [20].

Plant pathogenic microbes produce multiple effector proteins that are secreted into the plant apoplastic space or that are translocated into host cells by means of specialized translocation systems, such as type III secretion systems of Gram-negative bacteria [5]. Major functions of these effectors comprise suppression of host immunity and microbial accomodation in host tissues. Plant immunity-stimulating activities of effectors are mediated by immune receptors recognizing effector structures or effector-mediated manipulations of host targets [3], [5]. Likewise, phytopathogens preferring hemibiotrophic or necrotrophic lifestyles employ a wide range of structurally unrelated host-selective and host-nonselective toxins that are essential for establishment of infection [4]. As some effectors, some microbial toxins have been demonstrated to have dual functions in plant-microbe encounters as virulence factors and triggers of plant immunity [4], [21], [22], [23]. Toxin-mediated host immune activation is thereby supposed to be the result of host target manipulation or host cellular damage.

NLPs form a superfamily of proteins that are produced and secreted by bacterial, fungal and oomycete species [24], [25], [26]. NLPs have initially been discovered as cytotoxic proteins triggering leaf necrosis and plant defenses in dicotyledonous, but not in monocotyledonous plants [27]. 3D-structural analyses of *Pythium aphanidermatum* or *Moniliophthora perniciosa* NLPs, respectively, revealed substantial fold conservation with cytolytic, pore-forming actinoporins from marine organisms, suggesting that NLPs destabilize plant plasma membranes during infection thereby facilitating host cell death [4], [28]. Indeed, cytotoxic NLPs from the necrotrophic or hemibiotrophic phytopathogens *Pectobacterium carotovorum* pv. *carotovorum* (*Pcc*NLP), *Pythium aphanidermatum* (*Pya*NLP) or *Phytophthora parasitica* (*Pp*NLP) were shown to be key virulence factors sharing identical fold requirements for NLP phytotoxin and virulence activities [4]. Notably, NLP-mediated phytotoxicity and plant immune marker gene expression also required the same structural features. This finding together with the fact that the native 3D structure of NLP is required for its immunogenic activity, strongly supports the assumption that NLP-mediated plant cell necrosis results in the release of immunogenic DAMPs [4]. This process is reminiscent of microbial toxin-triggered inflammasome activation in vertebrates [29], [30].

There is accumulating evidence that NLP effectors have diversified in function [26]. The fungal pathogen *Mycosphaerella graminicola* produces *Mg*NLP that is toxic on dicot plants, but not on its monocot host, wheat [31]. Moreover, knock-down of a cytotoxic NLP in *Verticillium dahliae* resulted not only in reduced virulence on host plants, but also in reduced vegetative growth and conidiospore formation, suggesting a role of this NLP in asexual reproduction in addition to its role in fungal pathogenicity [32]. The biotrophic oomycete *Hyaloperonospora arabidopsidis* was shown to produce up to 10 NLP proteins all of which failed to cause necrosis in dicot plants including the host *Arabidopsis* [33]. Likewise, 11 of 19 *Phytophthora sojae* NLPs tested lacked phytotoxic activities [24]. Functional diversification among the two NLP subfamilies in this hemibiotrophic oomycete was further supported by the fact that genes encoding non-cytotoxic NLPs were expressed predominantly during early (biotrophic) phases of infection whereas cytotoxic NLP genes were expressed only at the onset of necrotrophic growth [24], [34], [35].

In this study, we have investigated plant immunogenic activities of NLP virulence factors in greater detail. Mutations that rendered *Pcc*NLP inactive with respect to cytotoxicity, host virulence and plant immune activation, also abolished the cytotoxic activity of another NLP (*Pp*NLP), but surprisingly left intact its ability to trigger plant defenses. This suggested the presence of another, yet unidentified immunogenic activity of *Pp*NLP. The elicitor activity of mutated *Pp*NLP could be pinpointed to a peptide fragment (nlp20) that triggered plant defenses in a manner comparable to that of bacterial flagellin. Importantly, immunogenic nlp20 fragments were found frequently in NLPs of bacterial, oomycete and fungal origin. In sum, we demonstrate that a common microbial effector harbors a PAMP motif that is found in both prokaryotic and eukaryotic microbes. Thus, its widespread occurrence is unique among microbial triggers of metazoan or plant innate immunity. In addition, the identification of two independent plant immunogenic mechanisms (PAMP- and toxin-induced immunity) within a particular microbial virulence factor is unprecedented and reveals an intricate complexity of microbial virulence and plant immune activation.

## Results

**Figure 1:**
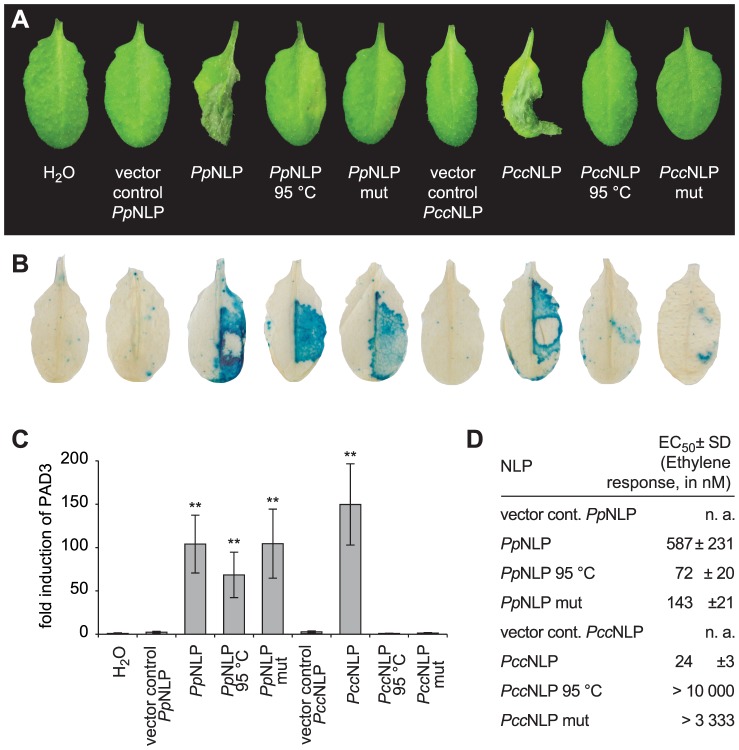
Cytotoxic and immunogenic activities of *Phytophthora parasitica* (*Pp*NLP) and *Pectobacterium carotovorum* (*Pcc*NLP) NLPs in *Arabidopsis*. Development of necrosis upon infiltration into leaves of 0.5 µM recombinant wild-type NLP, heat-treated NLP (1.5 hrs at 95°C) NLP or NLP mutant (mut) protein (H121A D124A). Infiltration of water or of protein preparations derived from expression systems transformed with insert-less vector served as controls. Pictures were taken 2 days post infiltration (**A**). *PR1::GUS* expression in transgenic plants upon infiltration of 0.5 µM recombinant NLP variants. GUS activity was histochemically visualized 24 hrs upon leaf infiltration (**B**). Treatments were the same as those shown in (**A**). *PAD3* gene expression in response to 0.3 µM recombinant NLP variants was quantified by qRT-PCR 4 hours after infiltration. *PAD3* transcript levels were normalized to those of EF1-α and are shown as fold induction compared to water control treatment. Bars represent mean ± SD of three replicates, asterisks mark significant differences to control treatments as determined by Student's t test, **P≤0.01 (**C**). Ethylene formation elicited by 0.5 µM recombinant NLP variants was quantified (EC_50_ values) in leaf discs 4 hrs upon infiltration. Numbers represent mean ± SD of three replicates. n.a., not applicable (**D**). All experiments shown were performed three times with similar results.

*Pectobacterium carotovorum* pv. *carotovorum*-derived *Pcc*NLP and *Phytophthora parasitica-*derived *Pp*NLP cause necrosis upon infiltration into leaves of *Arabidopsis thaliana* (Figure 1A) [4]. Heat treatment or simultaneous exchange of two highly conserved amino acid residues (H121A; D124A; positions correspond to those in *Pp*NLP) abolished necrotic (Figure 1A) and plasma membrane-permeabilizing activities (Figure S1A) of both proteins. Previously, the plant defense-stimulating activity of *Pcc*NLP had been linked to its cytotoxic activity, suggesting that toxin-mediated interference with host cell integrity triggered the release of yet unknown immunogenic damage-associated molecular patterns from lysed plant cells [4]. In support of this hypothesis, heat-denatured or mutant *Pcc*NLP failed to trigger plant defenses associated with PTI, such as *PR1::GUS* or *PAD3* gene expression and ethylene biosynthesis, whereas wild-type *Pcc*NLP triggered these responses (Figure 1 B–D, Figure S1B). In contrast, heat treatment of *Pp*NLP or mutated *Pp*NLP (H121A, D124A) did not affect plant defense-eliciting activity (Figure 1 B–D, Figure. S1B), suggesting that *Pp*NLP cytotoxicity may not solely explain its immunogenic potential.

### Identification and characterization of the immunogenic nlp20 motif within *Pp*NLP

**Figure 2:**
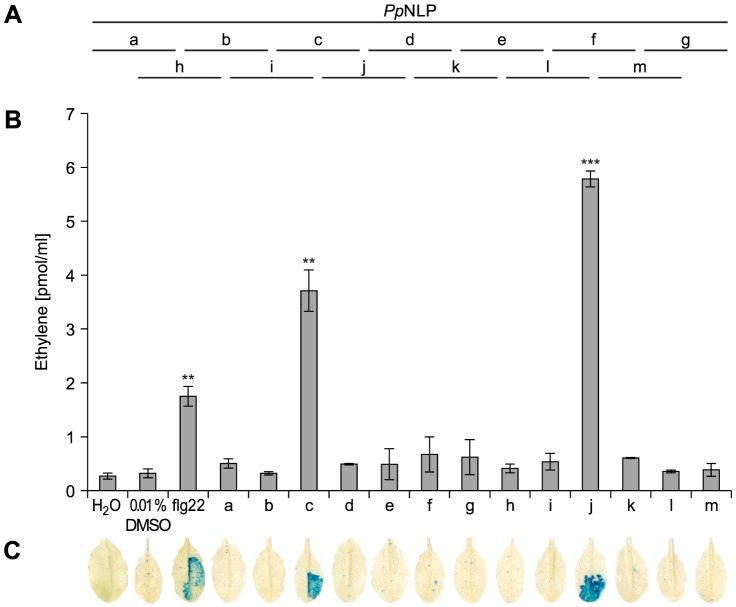
Immunogenic activities in *Arabidopsis* of synthetic *Pp*NLP sequence-derived peptides. Schematic representation of synthetic peptides (30-mer peptides, a–m) covering wild-type *Pp*NLP used for assessment of immunogenic activities (**A**). Ethylene formation elicited by 1 µM peptide (a–m) 4 hours upon leaf infiltration. Water, 0.01% DMSO (used to dissolve peptides) and flg22 treatments served as negative and positive controls, respectively. Bars represent means ± SD of three replicates. Asterisks mark significant differences to DMSO control treatments as determined by Student's t test, **P≤0.01, *** P≤0.001 (**B**). *PR1::GUS* expression upon leaf infiltration of 1 µM peptide solution. Histochemical staining was performed 24 hours after leaf infiltration (**C**). All experiments were performed in triplicate with similar results.

Typically, small epitopes within microbial patterns are sufficient for their immunogenic activities [2], [11], [19]. In search for such an immunogenic epitope within *Pp*NLP, nested synthetic peptides covering the entire *Pp*NLP protein sequence were produced and tested for their abilities to trigger ethylene production or *PR1::GUS* expression (Figure 2). Two overlapping peptides spanning residues G_84_ to V_129_ of *Pp*NLP (peptides c and j) proved both to be able to elicit plant defense-associated responses. These peptides share residues G_100_-D_113_ (GVYAIMYSWYFPKD, peptide 1, Table 1), suggesting that this fragment constitutes the core of the immunogenic activity of *Pp*NLP. Another set of nested synthetic peptides spanning the peptide 1 sequence were analyzed for their abilities to trigger ethylene production in *Arabidopsis* leaf disks. The EC_50_ value determined for peptide 1 was 322 nM (Table 1). N-terminal deletion peptides lacking residues G_100_-Y_106_ (GVYAIMY, peptide 4) or C-terminal deletion of residues K_112_ and D_113_ (peptide 2) substantially reduced elicitor activity, suggesting that both motifs are important for the immunogenic potential of *Pp*NLP (Table 1). In agreement with this, a peptide carrying an N-terminal extension, but lacking K_112_D_113_ (peptide 2) or peptides with C-terminal extensions, but lacking residues Y_102_-Y_106_ (peptides 4–6, peptide 12) were all inactive. Substantial N-terminal extension (peptide 8) did not increase elicitor activity of this peptide in comparison to peptide 1, suggesting that no further sequence information N-terminal of the G_100_-Y_106_ motif is required for elicitor activity of *Pp*NLP. To refine C-terminal sequence requirements for *Pp*NLP elicitor activity, we further tested peptides containing the Y_102_-Y_106_ motif or a fragment thereof (A_103_-Y_106_) and different C-terminal extensions beyond residues K_112_D_113_ (Table 1). These studies revealed two peptides with EC_50_ values of 14 (peptide 9) or 1,5 nM (peptide 13), respectively, as the most elicitor-active peptides, which are both substantially more active than peptide 1 (Table 1). As both peptides lack residue Y_102_ we conclude that it is dispensable for elicitor activity. In contrast, C-terminal extensions gradually enhance elicitor activities of the respective peptides, and together with motifs A_103_-Y_106_ and K_112_D_113_ constitute major determinants of *Pp*NLP immunogenic activity. Because of the origin of this motif from *Pp*NLP protein and because of the number of residues building peptides 9 and 13, these peptides were re-named nlp20 (*Pp*NLP) and nlp24 (*Pp*NLP), respectively. To identify amino acids within both peptides that are essential for their elicitor activities, an alanine-scanning mutagenesis was conducted (Table 1). Individual exchange of each amino acid by alanine (except A_103_W) identified residues I_104_, Y_106_, W_108_, and Y_109_, of which replacement reduced immunogenic activities of mutant peptides more than 1,000-fold as compared to nlp24 (*Pp*NLP). All other exchanges had significantly less or no effect on the activities of the mutant peptides (Table 1). Importantly, all of these residues are part of or are in close proximity to the A_103_-Y_106_ motif, highlighting again the importance of this motif for *Pp*NLP elicitor activity. Individual exchanges in the C-terminal regions of nlp20 (*Pp*NLP) or nlp24 (*Pp*NLP), respectively, affected immunogenic activities of the mutant peptides in a rather moderate manner.

**Table 1:** Identification of a minimum immunogenic epitope within *Pp*NLP.

Peptide No.	*Pp* NLP consensus sequence (amino acid residues 84–131)																																															EC_50_ ± SD [nM]
	G	S	G	Y	G	S	Q	V	Y	G	R	V	A	T	Y	N	G	V	Y	A	I	M	Y	S	W	Y	F	P	K	D	S	P	V	T	G	L	G	H	R	H	D	W	E	V	V	V	W	
c	G	S	G	Y	G	S	Q	V	Y	G	R	V	A	T	Y	N	G	V	Y	A	I	M	Y	S	W	Y	F	P	K	D																		101±34
j																	G	V	Y	A	I	M	Y	S	W	Y	F	P	K	D	S	P	V	T	G	L	G	H	R	H	D	W	E	V	V	V	W	34±10
1																	G	V	Y	A	I	M	Y	S	W	Y	F	P	K	D																		322±165
2														T	Y	N	G	V	Y	A	I	M	Y	S	W	Y	F	P																				16667±3055
3																			Y	A	I	M	Y	S	W	Y	F	P	K	D	S	P	V															217±65
4																								S	W	Y	F	P	K	D	S	P	V	T	G	L	G	H										>100000
5																													K	D	S	P	V	T	G	L	G	H	R	H	D	W	E					>100000
6																																		T	G	L	G	H	R	H	D	W	E	V	V	V	W	>100000
7											R	V	A	T	Y	N	G	V	Y	A	I	M	Y	S	W	Y	F	P	K	D																		84±11
8																	G	V	Y	A	I	M	Y	S	W	Y	F	P	K	D	S	P	V	T	G	L												482±45
9																				A	I	M	Y	S	W	Y	F	P	K	D	S	P	V	T	G	L	G	H	R									14±12
10																					I	M	Y	S	W	Y	F	P	K	D	S	P	V	T	G	L	G	H	R									133±10
11																						M	Y	S	W	Y	F	P	K	D	S	P	V	T	G	L	G	H	R									1550±328
12																							Y	S	W	Y	F	P	K	D	S	P	V	T	G	L	G	H	R									>50000
13																				A	I	M	Y	S	W	Y	F	P	K	D	S	P	V	T	G	L	G	H	R	H	D	W	E					1.5±0.7
14																								S	W	Y	F	P	K	D	S	P	V	T	G	L	G	H	R	H	D	W	E					>100000
15																				**W**	I	M	Y	S	W	Y	F	P	K	D	S	P	V	T	G	L	G	H	R									55±24
16																				A	**A**	M	Y	S	W	Y	F	P	K	D	S	P	V	T	G	L	G	H	R									1567±252
17																				A	I	**A**	Y	S	W	Y	F	P	K	D	S	P	V	T	G	L	G	H	R									42±9
18																				A	I	M	**A**	S	W	Y	F	P	K	D	S	P	V	T	G	L	G	H	R									1500±436
19																				A	I	M	Y	**A**	W	Y	F	P	K	D	S	P	V	T	G	L	G	H	R									18±10
20																				A	I	M	Y	S	**A**	Y	F	P	K	D	S	P	V	T	G	L	G	H	R									2833±777
21																				A	I	M	Y	S	W	**A**	F	P	K	D	S	P	V	T	G	L	G	H	R									2633±208
22																				A	I	M	Y	S	W	Y	**A**	P	K	D	S	P	V	T	G	L	G	H	R									5.4±3
23																				A	I	M	Y	S	W	Y	F	**A**	K	D	S	P	V	T	G	L	G	H	R									307±153
24																				A	I	M	Y	S	W	Y	F	P	**A**	D	S	P	V	T	G	L	G	H	R									89±31
25																				A	I	M	Y	S	W	Y	F	P	K	**A**	S	P	V	T	G	L	G	H	R									97±36
26																				A	I	M	Y	S	W	Y	F	P	K	D	**A**	P	V	T	G	L	G	H	R									57±28
27																				A	I	M	Y	S	W	Y	F	P	K	D	S	**A**	V	T	G	L	G	H	R									76±45
28																				A	I	M	Y	S	W	Y	F	P	K	D	S	P	**A**	T	G	L	G	H	R									30±11
29																				A	I	M	Y	S	W	Y	F	P	K	D	S	P	V	**A**	G	L	G	H	R									25±15
30																				A	I	M	Y	S	W	Y	F	P	K	D	S	P	V	T	**A**	L	G	H	R									23±7.6
31																				A	I	M	Y	S	W	Y	F	P	K	D	S	P	V	T	G	**A**	G	H	R									24±8
32																				A	I	M	Y	S	W	Y	F	P	K	D	S	P	V	T	G	L	**A**	H	R									220±78
33																				A	I	M	Y	S	W	Y	F	P	K	D	S	P	V	T	G	L	G	**A**	R									48±34
34																				A	I	M	Y	S	W	Y	F	P	K	D	S	P	V	T	G	L	G	H	**A**									283±40
35																				A	I	M	Y	S	W	Y	F	P	K	D	S	P	V	T	G	L	G	H	R	**A**	D	W	E					7.7±1.7
36																				A	I	M	Y	S	W	Y	F	P	K	D	S	P	V	T	G	L	G	H	R	H	**A**	W	E					6.2±5.6
37																				A	I	M	Y	S	W	Y	F	P	K	D	S	P	V	T	G	L	G	H	R	H	D	**A**	E					7.3±1.8
38																				A	I	M	Y	S	W	Y	F	P	K	D	S	P	V	T	G	L	G	H	R	H	D	W	**A**					7.3±1.7

Synthetic peptides derived from a *Pp*NLP fragment spanning amino acid residues 84–131 were tested for their abilities to trigger ethylene formation in *Arabidopsis* leaves. EC_50_ values were determined based upon using at least six different peptide concentrations. Peptides c and j are as indicated in Figure 2. Peptides 1–14 represent truncated versions of *Pp*NLP fragment 84–131. Peptides 15–38 are truncated versions of *Pp*NLP fragment 84–131 in which single amino acid residues are replaced by alanine residues (in bold). Data represent means ± SD of three replicates. Assays were performed three times with similar results.

To test whether the nlp20 (*Pp*NLP) motif derived from cytotoxic NLP would retain both immunogenic and cell death-causing activities, leaf necrosis and plasma membrane permeabilization assays were performed using equimolar concentrations of intact *Pp*NLP and of *Pp*NLP-derived nlp20 (*Pp*NLP) as well as 10-fold higher concentrations of the latter. As shown in Figure S1D–E, nlp20 (*Pp*NLP) failed to trigger either response, suggesting strongly that its immunogenic activity is not linked to cell death or plasma membrane disintegration. This conclusion is further supported by our findings that heat treatment or mutations within intact *Pp*NLP abolished its necrosis-inducing activity, but not its ability to trigger immunity-associated defenses (Figure 1A). Likewise, low nanomolar concentrations of nlp20 (*Pp*NLP) are required to trigger plant defenses (Table 1), which is in clear contrast to the failure of the peptide to trigger necrosis at 10 mikromolar concentrations (Figure S1D), again disconnecting nlp20 (*Pp*NLP)-induced defenses from the cytotoxic potential of intact *Pp*NLP.

### Immunogenic nlp20 patterns are widespread within diverse microbial lineages

NLPs are widespread microbial patterns that are found in bacteria, fungi and oomycete species [27], [36]. Inspection of NLP protein sequences from the various lineages revealed the presence of an nlp20-motif in numerous cases. To test whether nlp20-like peptides of NLPs from different microbial origins harbor PAMP activity, synthetic peptides representing bacteria- (*Bacillus subtilis*, *Bacillus halodurans*), fungus- (*Fusarium oxysporum*, *Botrytis cinerea*) or oomycete-derived (*Pythium aphanidermatum*) sequences orthologous to nlp20 (*Pp*NLP) were analyzed for their immunogenic potential. As shown in Table 2, all peptides tested exhibited the ability to trigger ethylene production, MAPK activation, production of reactive oxygen species (oxidative burst), *PR1::GUS* expression, and callose apposition (Figure S2A–E). For ethylene production, EC_50_ values were determined and found to be very similar for all nlp20 orthologs tested (Table 2). *Arabidopsis* seedling growth inhibition on agar plates containing flg22, elf18 or *At*Pep1 is a hallmark plant response to those patterns that are recognized by LRR-RK-type pattern recognition receptors. Remarkably, reduced seedling size in the presence of PAMPs was only detectable in flg22 control treatments, but not in cases when nlp20 (*Pp*NLP) or orthologous nlp20 peptides were tested (Table 2, Figure S2F). Likewise, *Phytophthora parasitica*-derived nlp20 failed to trigger an extracellular alkalinization response in *Arabidopsis* cell suspensions (Figure S2G).

**Table 2:** *Pp*NLP nlp20 orthologous peptides from bacterial, oomycete and fungal organisms exhibit comparable immunogenic activities in *Arabidopsis*.

Organism	Peptide	Peptide sequence	Ethylene production (EC_50_ in nM ± SD)	MAPK activation	Oxidative burst	*PR1::GUS* expression	Callose apposition	Growth inhibition
*Phytophthora parasitica*	nlp20 (*Pp*NLP)	AIMYSWYFPKDSPVTGLGHR	14±12	+	+	+	+	−
*Pythium aphanidermatum*	nlp20 (*Pya*NLP)	AIMYSWYMPKDSPSTGIGHR	8.9±2.7	+	+	+	+	−
*Fusarium oxysporum*	nlp20 (*Fo*NLP)	AIMYAWYWPKDQPADGNLVSGHR	43±13	+	+	+	+	−
*Botrytis cinerea*	nlp20 (*Bc*NLP)	AIMYSWYMPKDEPSTGIGHR	7.7±3.2	+	+	+	+	−
*Bacillus halodurans*	nlp20 (*Bh*NLP)	AIMYAWYFPKDSPSPGLGHR	5.7±2.4	+	+	+	+	−
*Bacillus subtilis*	nlp20 (*Bs*NLP)	AIMYSWYFPKDEPSPGLGHR	11±0.3	+	+	+	+	−

Synthetic peptides corresponding to nlp20 (*Pp*NLP) orthologous sequences from another oomycete, *Pythium aphanidermatum* (nlp20 (*Pya*NLP)), from fungi *Fusarium oxysporum* (nlp20 (*Fo*NLP)) and *Botrytis cinerea* (nlp20 (*Bc*NLP)), and bacterial species *Bacillus halodurans* (nlp20 (*Bh*NLP)) and *Bacillus subtilis* (nlp20 (*Bs*NLP)) were tested for their abilities to trigger ethylene formation (EC_50_ values), MAPK activation, production of reactive oxygen species (oxidative burst), *PR1::GUS* expression, deposition of callose and seedling growth inhibition. +, activation; −, no activation.

As shown in Figure 1, *Pcc*NLP mutants lacking cytotoxic activity also lacked immunogenic activity. In contrast, *Pp*NLP mutants devoid of cytotoxic activity remained immunogenic due to the presence of the nlp20 motif. In agreement with the apparent absence of an immunogenic nlp20 motif in *Pcc*NLP, a synthetic peptide derived from the *Pcc*NLP sequence that corresponds to the nlp20 motif in *Pp*NLP (GSFYALYFLK DQILSGVNSGHR), proved largely inactive with respect to activating ethylene formation, MAPK activation and *PR1::GUS* expression (Figure S3). Residual ethylene-inducing activity was observed for this peptide (EC_50_ 5520 nM), which was approximately 400 times less active than nlp20 (*Pp*NLP) (EC_50_ 14 nM) (Figure S3A).

### Sensitivity to nlp20 is restricted to particular plant families

**Figure 3:**
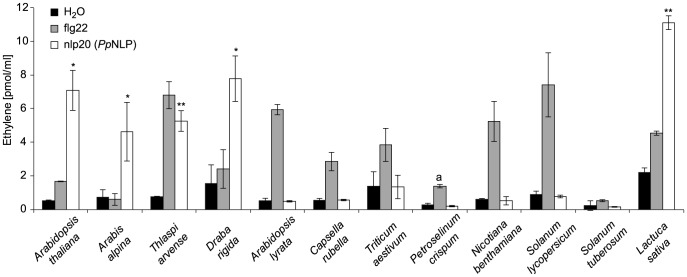
Analysis of nlp20 (*Pp*NLP) recognition systems in various plant families. Ethylene formation elicited by 1 µM synthetic nlp20 (*Pp*NLP) peptide 4 hours upon infiltration into leaves of the plant species indicated. Water and flg22 treatments served as negative and positive controls, respectively. Bars represent means ± SD of three replicates and asterisks mark significant differences to water control treatments as determined by Student's t test, *P≤0.05, **P≤0.01. Pep-13 from *P. sojae* transglutaminase was used as a positive control for *P. crispum* (a). Assays were performed in triplicate with similar results.

To analyze the relative distribution of nlp20 recognition systems among plants, we first tested whether other *Brassicaceae* species beside *Arabidopsis thaliana* responded to this peptide. As shown in Figure 3, *Arabis alpina*, *Thlaspi arvense*, and *Draba rigida* mounted an ethylene response to nlp20 (*Pp*NLP) treatment, suggesting that nlp20 recognition is widespread among the *Brassicaceae* family. Notably, another species from the genus *Arabidopsis*, *Arabidopsis lyrata*, did not respond to nlp20 (*Pp*NLP), but did so to the control treatment with flg22. Although surprising in the first place, this finding might just reflect that PAMP responsiveness is often even not entirely conserved among ecotypes of the same species. Neither solanaceous plants (tomato, potato, *Nicotiana benthamiana*) nor parsley (*Petroselinum crispum*, an *Apiaceae*) or wheat (*Triticum aestivum*, a monocotyledonous grass) responded to nlp20 (*Pp*NLP) (Figure 3). Failure to detect nlp20 (*Pp*NLP) responses in parsley is in agreement with our previous studies showing that this plant species lacks the ability to recognize NLP peptide fragments [37]. In contrast, ethylene production was detectable after treatment of leaves of lettuce (a member of the *Asteraceae* family) with nlp20 (*Pp*NLP) (Figure S4). As lettuce did not respond to an nlp20 (*Pp*NLP) derivative lacking PAMP activity in *Arabidopsis thaliana* (Figure S4) we conclude that nlp20 (*Pp*NLP) perception systems in both plants exhibit similar ligand specificities. Whether nlp20 recognition is even more widespread among plant families requires comprehensive, systematic surveys of its immunogenic activity.

### Nlp20 (*Pp*NLP) treatment primes plants for immunity to subsequent microbial infections

**Figure 4:**
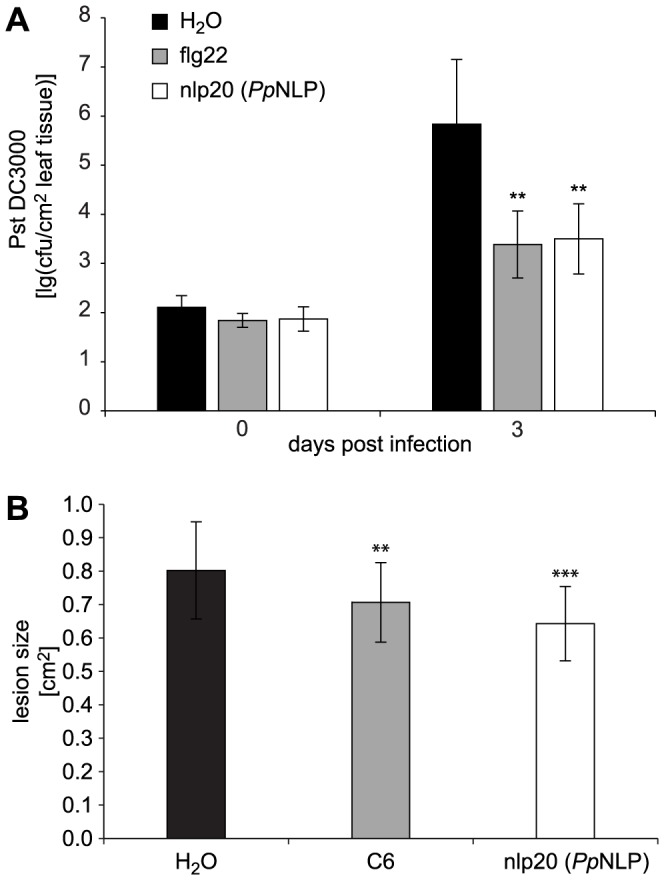
Treatment with nlp20 (*Pp*NLP) renders *Arabidopsis* less susceptible to bacterial and fungal infection. Leaves were infiltrated with 1 µM synthetic nlp20 (*Pp*NLP) 24 hours before inoculation of the same leaf with 10^4^ cfu ml^−1^ *Pseudomonas syringae* pv *tomato* strain DC3000 (*Pst* DC3000). Bacterial growth was determined at 0 and 3 days after leaf infiltration. Flg22 and water served as positive and negative controls respectively. Data represents means ± SD of six replicate measurements per treatment and data point. Asterisks indicate statistically significant differences to water control treatments (**P≤0.01, Student's t test). One of three independent experiments is shown (**A**). Leaves were pre-treated as in (**A**) before inoculation with 5 µl of a 5×10^6^ spores ml^−1^ of the fungus *Botrytis cinerea*. Treatments with chitin hexamer (C6) and water served as positive and negative controls, respectively. Data represent means ± SD of n = 28 plants per treatment. Asterisks indicate significant differences to water control treatments (**P≤0.01, ***P≤0.001, Student's t test) (**B**). Experiments were performed in triplicate with similar results.

PAMP treatment results in enhanced plant immunity to subsequent microbial infection [2], [11], [19]. For example, treatment with flg22 of *Arabidopsis* plants prior to infection with virulent *Pseudomonas syringae* pv. *tomato* DC3000 reduced bacterial growth by about 100-fold within three days post infection when compared to bacterial growth rates on mock-treated plants (Figure 4). Likewise, nlp20 (*Pp*NLP) treatment limited bacterial growth rates on ecotype Col-0 to a similar extent as did flg22 treatment, suggesting that both patterns have an immunogenic activity (Figure 4). Nlp20 (*Pp*NLP) also reduced bacterial growth on an *fls2 efr* genotype (Figure S5), thus ruling out flg22 contamination issues here that have raised concerns about recent studies on plant PRRs [38]. In contrast, pre-treatment with immunogenically inactive nlp20 (*Pcc*NLP) (Figure S3A) or peptide 20 (Table 1) did not result in reduced bacterial growth, which documents the ligand specificity of the observed biological phenomenon (Figure S5B). As further shown in Figure 4, nlp20 (*Pp*NLP) treatment also primed *Arabidopsis* plants for enhanced immunity to infection by the fungal phytopathogen *Botrytis cinerea*. Lesion sizes in plants pretreated were significantly smaller than those observed in mock-treated plants. Likewise, pre-treatment of lettuce with an nlp24 peptide derived from *H. arabidopsidis* nlp24 (*Ha*NLP3) enhanced resistance to infection with *Bremia lactucae* (Figure S6). Altogether, our findings demonstrate that nlp20 recognition contributes to plant immune activation and to reduced symptom development and microbial growth rates on infected plants.

## Discussion

Cytotoxic NLPs are microbial virulence factors facilitating both microbial infection and activation of plant immunity-associated responses. Toxin-mediated release of diffusible DAMPs from lyzed plant cells and subsequent PRR-mediated plant immune activation in neighboring cell layers or local systemic tissues has been proposed as the likely molecular mechanism underlying immunogenic activity of, for example, *Pcc*NLP [4]. Experimental findings in support of this model comprise (i) identical fold requirements for microbial virulence and immune activation, (ii) requirement of natively folded cytotoxic NLPs for immune activation and (iii) the apparent lack of NLP enzyme activity (no primary sequence or 3D-structure similarity to known enzymes). Other examples for microbial toxins as triggers of plant defenses include *Fusarium* spp.-derived fumonisin, *Phomopsis amygdali*-derived fusicoccin or *Cochliobolus victoriae*-derived, victorin. Toxin-induced immunity is thus considered a hallmark of innate immunity not only in metazoans, but also in plants [19], [29].

To our surprise, we have been able to unveil a second molecular mechanism by which cytotoxic NLPs are able to evoke plant immunity. This discovery was spurred by findings that mutations that rendered *Pcc*NLP non-cytotoxic and non-immunogenic failed to have the same effect in other cytotoxic NLPs, such as *Pp*NLP. We have now been able to identify a peptide motif (nlp20 motif) within *Pp*NLP and other NLPs that is missing in *Pcc*NLP. This strongly suggests that cytotoxic NLPs carrying the nlp20 motif are potentially capable of evoking plant immunity by two different mechanistic modes, by toxin action and by a classical PAMP motif. To our knowledge, this is an unprecendented finding as microbial patterns with dual immunogenic activities are currently unknown in both metazoan and plant immunity. These results shed light on how intricately complex and mechanistically diverse microbe sensing in individual plant microbe encounters might be. In support of this notion, *Arabidopsis thaliana* alone is capable of recognizing at least seven structurally different patterns derived of pseudomonads [10]. This and substantial diversification and expansion of gene families encoding plant PRRs strongly suggests that many more immunogenic patterns than those currently known might exist [14], [19]. Thus, the number of microbial patterns recognized in particular plant-microbe interactions together with different immunogenic modes of individual microbial patterns appears to represent an immunogenic potential of microbial surfaces of which complexity is most likely much larger than anticipated previously.

PAMPs triggering immunity in metazoans or plants are supposed to be widespread among microbial species [2], [39]. Bacteria-derived flagellin, peptidoglycans or lipopolysaccharides are patterns that are found across taxonomical orders. Likewise, fungus-derived chitin or oomycete-derived ß-glucan structures are extremely common among these organisms. The immunogenic nlp20 motif is unique, however, in that it is found conserved not only in NLPs of bacterial origin, but also in fungal and oomycete genera. To our knowledge, none of the currently known triggers of metazoan or plant innate immunity shows a comparably wide distribution pattern among eukaryotic and prokaryotic microbes. By using synthetic nlp20 peptides derived from two bacterial, oomycete and fungal organisms, respectively, we could demonstrate PAMP activity associated with NLPs from all three lineages. Currently, 1,091 NLP sequences can be retrieved from databases using the *Pp*NLP1 sequence as query (221, 558, 312 sequences of bacterial, fungal, oomycete origin, respectively). Preliminary inspection of these sequences for the presence of the nlp20 motif and of those residues that are crucial for its PAMP activity (I_104_, Y_106_,W_108_, Y_109_) revealed that a remarkably low number of bacterial sequences (20 out of 221), but a majority of fungal and virtually all oomycete NLPs likely contain an elicitor-active nlp20 motif. In sum, this motif is a predominant feature within a vast number of NLP sequences particularly in eukaryotic NLP-producing microorganisms. Importantly, in comparison to the relatively small numbers of NLP-encoding genes in fungal genomes, the number of NLP genes has expanded significantly in oomycete species. For example, the *P. sojae* genome harbors 33 NLP genes 20 of which have been shown to be expressed, whereas *H. arabidopsidis* encodes 12 NLP genes 8 of which are expressed early during plant infection [24], [33]. Clustering of these sequences in species-specific groups and the occurrence of non-cytotoxic NLPs indicates rapid expansion and functional diversification within these gene families without an apparent deleterious effect on the nlp20 motif. Predictions whether bacterial NLPs have largely lost this motif during evolution (such as phytopathogenic *P. carotovorum*) or whether nlp20 motif-containing NLPs have been acquired from eukaryotic species via horizontal gene transfer are difficult to make as of now.

The nlp20 motif exhibits molecular features similar to that of the prototype immunogenic pattern, bacterial flagellin (flg22) [2]. It is active at low nanomolar concentrations, it triggers several immunity-associated plant responses including broad spectrum immunity to bacterial and fungal infection, and it is evolutionarily conserved within NLPs. Although flagellin and nlp20 patterns trigger a set of overlapping plant responses, substantial differences are apparent, too. For example, flg22 evokes extracellular alkalinization in *Arabidopsis* cell suspensions and *Arabidopsis* seedling growth retardation [40], [41], whereas nlp20 does not trigger these responses. Whether or not these differences in the immunogenic activities of both patterns reflect recognition by different receptor types remains to be seen.

In summary, we here report the identification of a common immunogenic pattern within a microbial virulence factor. The nlp20 motif of bacterial, fungal or oomycete NLPs possesses the ability to trigger plant immune responses in a manner comparable to bacterial flagellin. Unique features of this pattern comprise (i) its presence in both prokaryotic and eukaryotic microbes and (ii) the fact that it constitutes a second immunogenic principle within cytotoxic NLPs. Further, we suggest that a microbial effector might have driven the emergence of plant pattern recognition systems mediating PTI. This is important as it is reminiscent of the evolution of immune receptors mediating recognition of pathogen race-specific microbial effectors and activation of ETI [3], [5], [6]. In this respect, our findings support the concept of an evolutionary and functional continuum between plant PTI and ETI [20].

## Materials and Methods

### Plant materials and growth conditions

*Arabidopsis* Col-0 and *efr fls2* plants were grown in soil at 22°C, 8 h light and used for the experiments at an age of 5–6 weeks. Plants used for infection assays were grown under translucent cover.

### Pathogenicity assays

5–6 weeks old *Arabidopsis thaliana* Col-0 plants were primed 24 hours before bacterial or fungal infection by leaf infiltration of nlp20 (*Pp*NLP), flg22, C6 (1 µM peptide solution) or mock-treatment, respectively. To assess bacterial growth rates, *Pseudomonas syringae* pv. *tomato* DC3000 (*Pst* DC3000) strain was used. The strain was maintained at 28°C on King's B medium (20 g l^−1^ glycerol, 40 g l^−1^ proteose pepton, 15 g l^−1^ agar) containing rifampicin and cycloheximide (50 µg ml^−1^). Overnight cultures were centrifuged, washed twice in 10 mM MgCl_2_ and adjusted to a bacterial density of 10^4^ cfu ml^−1^. Primed leaves were pressure-infiltrated with the bacterial solution and the plants were kept under high humidity. Leaves were harvested and surface sterilized in 70% EtOH and ddH_2_0 for 1 minute each. Two leaf discs per plant were stamped out, ground in 10 mM MgCl_2_, diluted serially 1∶10 and plated on LB plates containing the appropriate antibiotics. After 2 days of incubation, colony-forming units were counted. For fungal infection, primed *Arabidopsis* leaves were drop-inoculated with 5 µl droplets of *Botrytis cinerea* isolate BO-10 containing 5×10^6^ spores ml^−1^ in PDB (potato dextrose broth, Sigma) and kept under high humidity. Photographs were taken 2 days after infection and lesion sizes were determined using the Photoshop CS6 Lasso tool. Selected pixels were counted and the lesion size in cm^2^ was calculated using a 0,5 cm^2^ standard. For oomycete *Bremia lactucae* infection, *L. sativa* leaf discs were vacuum-infiltrated with 1 µM nlp24 (*Ha*NLP3) and 24 hours later treated with a 20 µl droplet spore suspension (120 spores/µl). Sporulation was assessed 8 days post inoculation.

### Recombinant protein expression and purification

For functional studies, secretory expression of NLPs was performed either in *Pichia pastoris* GS115 (secretory expression plasmid pPIC9K, Multi-Copy Pichia Expression Kit Instructions, Invitrogen) or in the NLP-deficient *Pectobacterium carotovorum* subsp. *carotovorum* SCC3200 strain (*Pcc nlp^−^*). Isolation of *Pcc*NLP proteins from the periplasmic space of transgenic *Pcc nlp^−^* was performed by osmotic shock as described [42]. Purification of *Pp*NLPs from *P. pastoris* culture medium or from *P. carotovorum* subsp. *carotovorum* SCC3200 periplasmic protein solution was achieved by ion exchange chromatography followed by gel filtration (GE Healthcare). As ion exchanger either HiTrap Q FF (equilibrated in 20 mM Tris-HCl pH 8.5: *Pp*NLP) or HiTrap SP FF (equilibrated in 50 mM MES pH 5.7: *Pcc*NLP) was used. Following elution (0–500 mM KCl in equilibration buffer), NLP containing fractions were pooled and subjected to HiLoad™ 16/60 Superdex 75, equilibrated in 150 mM KCl in the corresponding buffer. NLP containing fractions were finally pooled and dialyzed against H_2_O. Protein concentrations were calculated by UV spectroscopy (wavelength λ_280_) using the protparam tool (http://web.expasy.org/protparam) to determine protein-specific extinction coefficients ε_280_ for each protein. Determinations were verified by SDS-PAGE using a standard protein solution.

### Synthetic peptides

Peptides were purchased from Genscript Inc., prepared as 10 mM stock solutions in 100% DMSO, and diluted in water prior to use. DMSO concentrations corresponding to those in peptide solutions used in this study did not trigger themselves any of the responses shown here.

### Plant immune responses

For MAPK activity assays, infiltrated plant material was harvested after 15 minutes and frozen in liquid nitrogen before used for protein extraction in 20 mM Tris-HCl pH 7.5, 150 mM NaCl, 1 mM EDTA, 1% Triton X-100, 0,1% SDS, 5 mM DTT, Complete Protease Inhibitor Mini, EDTA-free (Roche, Mannheim), PhosStop Phosphatase Inhibitor Cocktail (Roche, Mannheim). After pelleting the cell debris (10 min, 16000 g, 4°C), the supernatant (30 µg protein) was separated on a 10% SDS-PAGE and transferred to a nitrocellulose membrane and activated MAPK6, 3 and 4 were detected by western blotting using the anti phospho p44/42-MAPK antibody from rabbit (Cell Signaling Technology, The Netherlands). For ROS burst measurements two leaf pieces, floated on ddH_2_O overnight, were placed in one well of a 96-well plate, containing 100 µl of a 20 µM L-012 and 0.5 µg ml^−1^ peroxidase solution. Background was measured shortly in a 96-well Luminometer, (Mithras LB 940, Berthold Technologies) before elicitation with a peptide solution or control treatment respectively. The detection of ethylene was performed as described [40]. Leaf pieces were incubated in 20 mM MES buffer, pH 5.7. To visualize callose apposition, leaves were treated as described [40] and harvested 24 hours after infiltration of a peptide solution. Quantification of callose was performed by counting selected pixels and calculated in % relative to the respective image section of the leaf surface. Pictures were analyzed using Photoshop CS6 Magic tool, hereby removing background and leaf-veins within a certain color range. (Use: white, Mode: normal, Opacity: 100%). Medium alkalinization in suspension-cultured *Arabidopsis* cells and detection of GUS enzyme activity in *PR1::GUS* transgenic *Arabidopsis* plants were performed as described previously [40], [43]. Surface-sterilized *Arabidopsis* Col-0 seeds were grown in ½ MS liquid medium supplemented with 1 µM of nlp20 (*Pp*NLP) peptide or its orthologs respectively, and flg22 or H_2_O serving as controls. Root length of two weeks-old seedlings was determined upon transfer onto agar plates.

### RNA isolation and RT-PCR

*Arabidopsis* leaves were infiltrated with 300 nM *Pp*NLP or *Pcc*NLP, heat-denatured (1.5 hours, 95°C) proteins or mutant versions (H121A D124A), respectively. RNA was isolated using the RNeasy Plant MiniKit (Qiagen) and synthesis of cDNA was performed by means of the RevertAidTM MuLV reverse transcriptase (Fermentas). Quantitative real-time PCR amplification was carried out in the presence of SYBR Green (Bio-Rad) with an iQ5 iCycler (Bio-Rad). Amplification of EF1-α served as internal standard. Data were analyzed according to the 2^−ΔΔCT^-method [44]. Gene induction (fold change) by NLPs was presented as the average of 3 determinations plus or minus standard deviation relative to the expression level of H_2_O infiltration.

### Calcein release of plasma membrane vesicles

Calcein release from intact *Arabidopsis* plasma membrane vesicles was performed as described [4].

## Supporting Information

Figure S1 **Comparison of cytotoxic and immunogenic activities of** ***Pp*** **NLP,** ***Pcc*** **NLP and nlp20 (** ***Pp*** **NLP).** Calcein release induced by *Pp*NLP, *Pcc*NLP or nlp20 (*Pp*NLP) from purified plasma membrane vesicles prepared from *Arabidopsis thaliana* leaves. Vesicles were treated with either 333 nM wild-type NLP, heat-treated NLP (95°C), mutant (mut) NLP or nlp20 (*Pp*NLP) peptide. Calcein release is calculated as the percentage of the maximum release as determined by addition of Triton X-100 at the end of the assay (**A**). Ethylene formation triggered upon *Arabidopsis* leaf infiltration of different concentrations of wild-type, heat-treated (95°C), mutant (mut) NLP variants (**B**). Data points represent n = 3 repetitions, and one representative experiment of three is always shown. Image of a Coomassie-stained SDA-PAGE gel documenting the purity of recombinant proteins used (**C**). Leaf necrosis (**D**) and calcein release (**E**) triggered by nlp20 (*Pp*NLP) peptide. One representative experiment of three is shown (**C–E**).
(EPS)

Figure S2 **Immunity-associated responses in Arabidopsis elicited by nlp20 (** ***Pp*** **NLP) and its orthologs.** Ethylene formation (**A**), MAPK activation (**B**), production of reactive oxygen species (**C**), *PR1::GUS* expression (**D**) and callose apposition (**E**) were determined upon infiltration into leaves of 100 nM nlp20*Pp* orthologous peptides derived from microorganism given in Table 2. Water and flg22 treatment served as controls. Panels (**A**) and (**C**) share the same color code. In (**E**), the diagram depicts callose apposition in % ± SD of three image sections of the leaf surface, counted as pixels. Photographs show the microscopic images after clearing callose depositions from background and leaf-veins. *Arabidopsis* seedlings were grown for two weeks under short day conditions in liquid ½ MS medium supplemented with 1 µM nlp20*Pp* peptide, its orthologs, or flg22 as a positive control respectively, and root length was determined after transfer onto agar plates. The upper panel documents quantification of 3 representative seedlings shown in the lower panel (**F**). *Arabidopsis* cell suspensions were supplemented with the nlp20*Pp* concentrations indicated or 100 nM flg22 and changes in extracellular pH were monitored continuously (**G**). All assays were performed in triplicate with similar results using the protocols described in Materials and Methods. One of three experiments is shown.
(EPS)

Figure S3 **Nlp20 (** ***Pcc*** **NLP) does not induce defense responses in** ***Arabidopsis*** **.** Elicitation of ethylene formation (**A**), MAPK activation (**B**) and *PR1::GUS* expression (**C**) in leaves infiltrated with peptide concentrations as indicated (**A**) or 100 nM (**B, C**). Peptides used were nlp20 (*Pp*NLP) (closed circles) and nlp20 (*Pcc*NLP) (GSFYSLYFLKDQILNGVNSGHR, open circles). (**B**) Activation of MAPK6, 3 and 4 detected 15 minutes after leaf infiltration as visualized detected by anti p44/p42 antibody staining. Ponceau S staining served as a loading control. (**C**) For *PR1::GUS* expression analysis, leaves were harvested 24 hours after treatment and stained histochemically. One of three experiments is shown.
(EPS)

Figure S4 ***Lactuca sativa*** **recognizes nlp20 (** ***Pp*** **NLP).** Leaf pieces of *Lactuca sativa* were infiltrated with 1 µM nlp20 (*Pp*NLP) or an inactive variant (peptide 20, Table 1) and ethylene formation was determined. Treatments with flg22 or water served as positive and negative controls, respectively. Bars represent mean ± SD of three replicates and asterisks mark significant differences to water control treatments as determined by Student's t test, *P≤0.05, **P≤0.01.
(EPS)

Figure S5 **Treatment with nlp20 (** ***Pp*** **NLP) renders** ***Arabidopsis efr fls2*** **less susceptible to bacterial infection (A), but elicitor-inactive nlp20 derivatives fail to prime plants for immunity to subsequent infection (B).** Leaves were infiltrated with 1 µM synthetic nlp20 (*Pp*NLP) (**A**), nlp20 (*Pcc*NLP) or peptide 20 (see Table 1) (**B**) 24 hours before inoculation of the same leaf with 10^4^ cfu ml^−1^ *Pseudomonas syringae* pv *tomato* strain DC3000 (*Pst* DC3000). Bacterial growth was determined at 0 and 3 days after leaf infiltration. Flg22 and water served as positive and negative controls respectively. Data represent means ± SD of six replicate measurements per treatment and data point. Asterisks indicate statistically significant differences to water control treatments (**P≤0.01, ***P≤0.001, Student's t test). One of three independent experiments is shown.
(EPS)

Figure S6 **Treatment with** ***Hyaloperonospora arabidopsidis*** **nlp24 (** ***Ha*** **NLP3) renders** ***Lactuca sativa*** **less susceptible to the oomycete,** ***Bremia lactucae*** **.** *L. sativa* cv. Olof leaf discs were vacuum-infiltrated with 1 µM nlp24 (*Ha*NLP3) 24 hrs prior to inoculation with 20 µl of a *B. lactucae* isolate Bl:24 spore suspension (120 spores/µl). Oomycete sporulation was assessed 8 days post inoculation. Asterisks indicate statistically significant differences to water control treatments ***P≤0.001, Student's t test). Experiments were repeated in triplicate with similar results.
(EPS)
